# Mechanical Properties of Injection Molded PP/PET-Nanofibril Composites and Foams

**DOI:** 10.3390/polym14142958

**Published:** 2022-07-21

**Authors:** Lun Howe Mark, Chongxiang Zhao, Raymond K. M. Chu, Chul B. Park

**Affiliations:** 1 Microcellular Plastics Manufacturing Laboratory, Department of Mechanical and Industrial Engineering, University of Toronto, Toronto, ON M5S 3G8, Canada; lhmark@mie.utoronto.ca (L.H.M.); zhaocx@mie.utoronto.ca (C.Z.); raymond.chu@sabic.com (R.K.M.C.); 2 SABIC Limburg B.V., 6167 RD Geleen, The Netherlands

**Keywords:** fiber reinforced thermoplastics, nano composites, foam injection molding, fibrillated nanofibers

## Abstract

The creation and application of PET nanofibrils for PP composite reinforcement were studied. PET nanofibrils were fibrillated within a PP matrix using a spunbond process and then injection molded to test for the end-use properties. The nanofibril reinforcement helped to provide higher tensile and flexural performance in solid (unfoamed) injection molded parts. With foam injection molding, the nanofibrils also helped to improve and refine the microcellular morphology, which led to improved performance. Easily and effectively increasing the strength of a polymeric composite is a goal for many research endeavors. By creating nanoscale fibrils within the matrix itself, effective bonding and dispersion have already been achieved, overcoming the common pitfalls of fiber reinforcement. As blends of PP and PET are drawn in a spunbond system, the PET domains are stretched into nanoscale fibrils. By adapting the spunbonded blends for use in injection molding, both solid and foamed nanocomposites are created. The injection molded nanocomposites achieved increased in both tensile and flexural strength. The solid and foamed tensile strength increased by 50 and 100%, respectively. In addition, both the solid and foamed flexural strength increased by 100%. These increases in strength are attributed to effective PET nanofibril reinforcement.

## 1. Introduction

In situ fibrillated composites can be used to overcome many the challenges of long-aspect ratio reinforcements [1]. With many long-aspect ratio fibers, the high shear needed for dispersing and distributing the fibers can have detrimental effects upon the reinforcing fibers themselves. Fiber breakage will often result in short fibers and lower mechanical performance when compared to properly compounded long-aspect fibers. Using fibrillated reinforcements, several of these drawbacks can be circumvented. By distributing and dispersing an immiscible domain before fibrillation, long-aspect ratio fibrils are not broken as they are not yet formed [2]. The production of in situ fibrillated materials have been studied using several methods, however, their applications in subsequent processes have rarely been studied.

In situ fibrillated reinforced composites have garnered a great deal of attention ever since their advent [3,4,5]. In situ fibrillated composites refer to a class of composites where the secondary, reinforcing phase of the fibril shape is created within the primary matrix [6]. By combining two immiscible polymers together and applying high extensional stresses, high strength composites can be achieved. Primarily uniaxial extensional stresses have been used, but shear and planar extensional stresses have also been used [7]. New and innovative polymeric composites that are stronger, lighter, and more functional are always in demand in both engineered and consumer goods. The ability to produce parts with lower costs will increase the profits for the manufacturer. Keeping the life cycle and final disposal of the polymer composites as design considerations is important to combat the waste and environmental impacts [8,9].

In this work, the term fiber refers to the shape of the primary PP matrix after spunbonding, is typically a few microns in diameter, and is a long continuous strand [10]. The term fibril refers to the secondary micro- or nano-sized fibrillated PET found within the fiber (primary matrix).

As previously mentioned, there are several distinct advantages over traditional methods of fiber reinforcement in the areas of dispersion, distribution, and fiber breakage. While compounding traditional fillers (e.g., nanoclay, glass fibers, CNTs, etc.), high shear is often used to ensure that the filler is distributed thoroughly throughout the matrix by intensive mixing [11,12]. Moreover, the high shear compounding is also used to breakdown large filler agglomerations to increase dispersion [13]. However, due to the high shear used, it will induce the severe breakage of fiber fillers due to mechanical degradation [14]. Unfortunately, fiber breakage is detrimental to the mechanical properties as the reinforcement performance is lowered. By fibrillating the fibers in situ within the matrix itself, these drawbacks can be overcome. Because the fibrillation stage is decoupled from the compounding stage, the micro/nano-fibrils are not yet formed, and thus, there is no risk of breakage occurring [5].

Typically, creating an in situ fibrillated composite consists of three stages: (1) compounding, (2) drawing, and (3) isotropization or end-use application [15]. The matrix and reinforcement are blended in a twin-screw compounder (TSE) and a sea-island morphology is created when their respective proportions are suitable [16]. Adding excessive amounts of the reinforcement can often result in a co-continuous blend. To create the smallest fibrillated fibrils, the sea-island morphology should have domains (islands) that are small and uniformly dispersed [16,17]. Having a proper compounding stage lays the foundation for better results downstream in the fibrillation process. With in situ fibrillation, the materials are selected so that they have differing melting temperatures (T_m_), with the reinforcement melting temperature (T_m,Rei_) 40 °C above the matrix melting temperature (T_m,Mat_). This temperature difference allows for the reinforcement polymer to be melted while compounding, but rigid during the drawing and isotropization stages.

To fibrillate the reinforcement, the sea-island blend undergoes a strong bulk deformation, for which typically uniaxial extensional stresses are used. Uniaxial stress is among the easiest, simplest, and most effective methods to transform the blends into fibers. While experiencing the extensional stress, the matrix becomes deformed and elongated; within the matrix, the stresses are also transmitted to the domains. As time passes, the domains become deformed in the stress direction and small tendrils appear from the domains. With sufficient time, the domains are fibrillated and can coalesce together with their neighbors to create longer fibrils. Melt spinning [17], spunbonding [18], melt blowing [19], and extrusion drawing [20,21] are common processes for fibrillation. Extrusion drawing is commonly used among research settings as the additional infrastructure or machine requirements are lower. Melt spinning, spunbonding, and melt blowing are typical processes used for creating different forms of yarns or non-woven textile fabrics. Each process integrates uniaxial extensional forces in their processes, which makes them ideal candidates for creating in situ fibrillated fibers. 

Isotropization is one of the last stages of fibrillated composite creation; it serves to randomly orient fibrils and decrease anisotropy. During the process, the temperature is lower than the T_m,Rei_ to avoid melting or otherwise damaging the fibrils. In laboratory/research settings, hot compression molding is often used to create test specimens. However, to apply this type of material to a more industrial environment, the materials need to be reconfigured into pellets for continuous processes such as extrusion [2] and injection molding [22]. Some reinforced fibers can also be used as-is, skipping a reprocessing stage before isotropization. The textile materials produced via filaments from melt spinning, or via nonwoven fabrics from melt blowing and spunbonding can potentially be directly fed into a machine. After spunbonding, the fibers are collected and roll calandered together to create non-woven materials. The calandered materials have sufficient tear strength to be collected as a spool on a core. This spool can be used to feed the injection molding machine, avoiding the need to have to pelletize the material. 

As previously mentioned, for nanofibril composites, the two polymeric components were selected to have distinct and separate melting temperatures. Being able to later reprocess the composite while still maintaining the nanofibril morphology is vital to ensuring high reinforcement properties. Earlier internal testing showed that during isotropization, a high processing temperature or high processing rates can degrade the fibrils. As the fibrils are heated during subsequent processing, they are subjected to excessive heat and stress, which can allow them to soften, relax, and distort. When degraded, the fibrils can shrink back from their elongated morphology, resulting in a low-aspect ratio, larger diameter domains that can be fibrillar or ellipsoidal.

PP composites reinforced with nanofibril PET have been produced using a variety of methods in lab scale settings. However, their application in continuous pilot or industrial processes has not been extensively investigated. Therefore, it is of interest to find methods and strategies to easily create these nanofibrillated materials. In this work, spunbonding was implemented to generate fibrillated composites. The spunbond method has several advantages including the ease of production with a high drawing ratio/extensional force using a vacuum attenuator. This attenuation provides an extra quenching process that allows for faster solidification of the fiber and an increase in the degree of molecular orientation. In this work, we demonstrated a method to easily create PET nanofibril composites and then applied them for solid and foam injection molding. The tensile and flexural properties of both the solid and foam composites were tested. 

## 2. Materials and Methods

### 2.1. Materials

A polypropylene (PP) grade (PP3155) from ExxonMobil (Irving, TX, USA) with an MFR of 36 g/10 min (at 230 °C/2.16 kg) was selected as the matrix/microfiber material. A polyethylene terephthalate (PET) grade (HOT) from LOTTE Chemicals (Seoul, Korea) was used as the reinforcement fibril material. The PET had an intrinsic viscosity of 0.78 dL/g. Both the PP and PET grades were homopolymers. The melting points of the neat PP and PET were 166.0 and 257.7 °C, respectively, as determined by differential scanning calorimetry (DSC) (see Section 2.4). The carbon dioxide gas used during the foam injection molding was supplied by Messer Canada (Mississauga, ON, Canada).

### 2.2. Spunbond Sample Preparation

**Figure 1:**
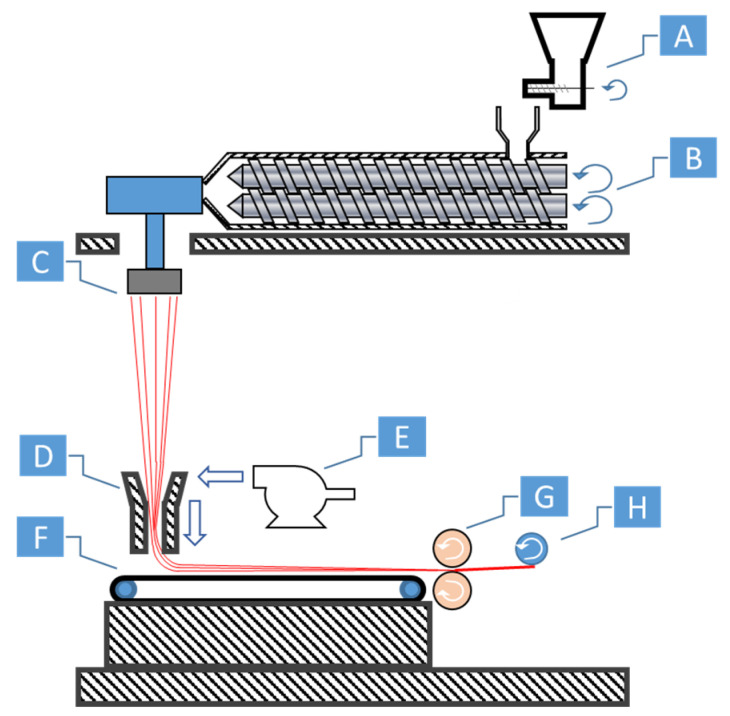
Spunbond machine: (**A**) feeder, (**B**) twin-screws, (**C**) spinneret, (**D**) drafter/attenuator, (**E**) blower, (**F**) conveyer, (**G**) calandering rollers, and (**H**) wind up.

For all cases, the PP or the composite materials were dried prior to usage at 80 °C in a vacuum oven to remove any moisture. The PET pellets were dried at 120 °C in a vacuum oven before their first processing stage. The samples were prepared using a spunbond machine (LCWF-MS-01, Yantai Langcai Plastic Technology Co., Ltd., Zhaoyuan City, Shandong, China) equipped with a TSE (see Figure 1). The processing parameters are shown in Table 1.

In addition, Table 2 lists the PET loadings that have been investigated and their material code for this paper. The SP/100/0 is a control sample with the same processing history, but without any PET reinforcement. The SP/AR control sample was created later during injection molding using the as-received PP for comparison.

The spunbond process is shown in Figure 1. The PET and PP pellets were compounded together in the TSE (Figure 1B), spunbonded (Figure 1D), and then calandered together (Figure 1G). The spunbonding process uses a vacuum drafter/attenuator to elongate the fibers from the spinneret. After the fibers exit the drafter and land onto the conveyer, the fibers are sintered together via a pair of heated calandering rollers, which creates a non-woven fabric. After calandering, the fabric web is wound onto a cylindrical cardboard core (Figure 1H). The rolls of calandered spunbond fabric had sufficient basis weight and strength to be directly fed into the injection molding machine later (see Section 2.5.1). 

### 2.3. Nanofibril Structure Characterization 

The nanofibrils were investigated after spunbonding. To measure the fibrils, the surrounding PP matrix was completely etched away using xylene at 120 °C. After drying and rinsing the sample using acetone, the remaining PET nanofibrils were mounted onto a SEM stub for viewing. 

To view the fibrils, the samples were sputter coated (SC7610, Quorum Technologies, Laughton, UK) with platinum under an argon atmosphere. A Phenom Pro SEM (Thermo Fisher, Waltham, MA, USA) was used to image the fibers and nanoscale fibrils using a voltage of between 3 and 5 kV. The fibers or fibrils in the SEM images were analyzed using ImageJ, and more than 100 fibers or fibrils were analyzed for each condition.

### 2.4. DSC Characterization

A differential scanning calorimeter (DSC 250, TA Instruments, New Castle, DE, USA) was used to analyze the thermal behavior of the composites and the influence of the PET nanofibrils. After spunbonding, the fibers were cold pressed (room temperature) together into 16 mm diameter by 2 mm thick disks. The cold press compacts the macrofibers together and increases the bulk density, giving the disks smooth top and bottom surfaces while preserving the thermal history. From the cold pressed disk, a DSC sample between 10 and 15 mg was cut and sealed within aluminum Tzero pans. The samples were then run on a heat–cool–heat cycle with a ramp of 10 °C/min under a nitrogen atmosphere. 

### 2.5. Injection Molding

To evaluate how the PET nanofibrils reinforced the composite part, mechanical testing was performed on samples cut from the injection molded (IM) test plaques. Both the solid (unfoamed) (IM) and foam IM (FIM) samples were tested for their tensile and flexural properties.

#### 2.5.1. Solid (Unfoamed) Injection Molding

To create the final parts and examine the use of nanofibril PET materials, the spooled rolls of spunbonded materials were placed into a 50-ton 270C ALLROUNDER (ARBURG, Loßburg, Germany) injection molding (IM) machine. This IM machine was used to create both the solid (unfoamed) and foamed samples. To create microcellular foam IM (FIM) parts, a MuCell SCF II (Supercritical Fluid) delivery system (Trexel Inc., Wilmington, MA, USA) was attached.

**Figure 2:**
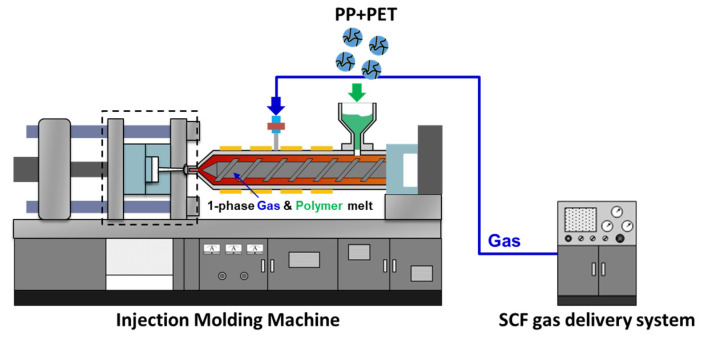
The Arburg injection molding machine.

Figure 2 shows the IM machine and Table 3 shows the relevant processing parameters. As previously mentioned, the barrel temperature and the screw speed are kept low to reduce any shear heating or fibril breakage that could lead to fibril degradation.

**Figure 3:**
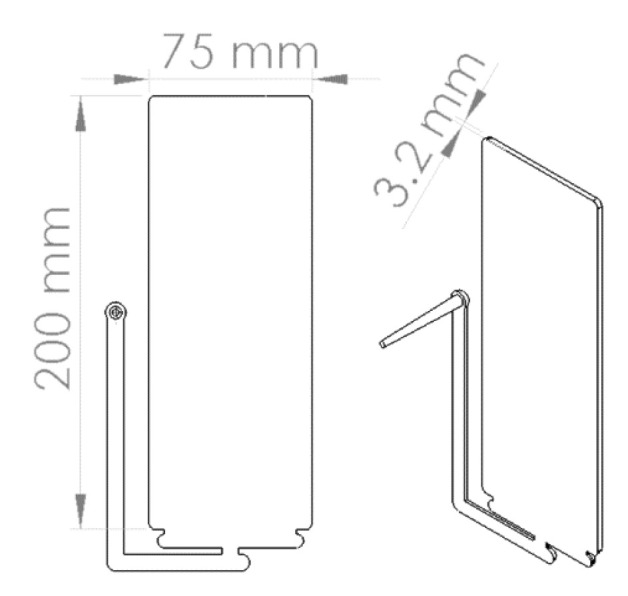
The test plaque dimensions.

The injection mold has a custom cavity built to allow for longer parts on a shorter injection molding machine. The mold cavity can accommodate ASTM D638 Type 1 bone shaped specimens and the flexural testing of thicker foam samples. Figure 3 shows the dimensions of the mold cavity (200 × 75 × 3.2 mm, 8.0 × 3.0 × 0.125”) cavity. The cavity is capable of high pressure and low pressure foam injection molding. 

**Figure 4:**
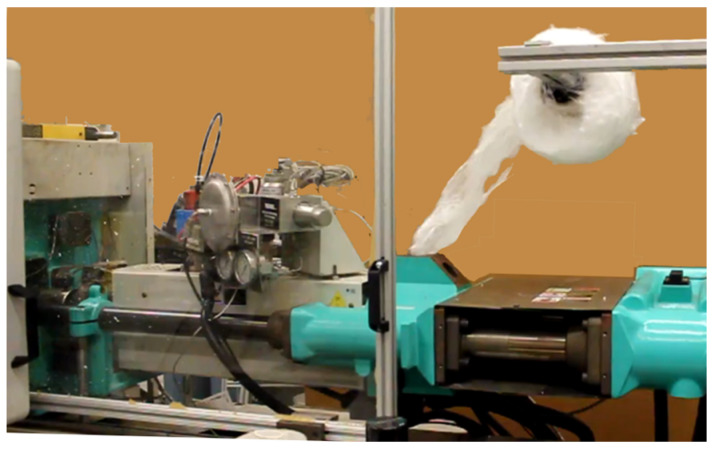
Feeding the injection molding machine.

As previously discussed, after spunbonding, the fibers are calandered together and collected as large spools of fabric webs. These spools are then mounted onto the Arburg IM and the webs were directly loaded into the IM machine. Using the IM screw, the web catches in-between the screw and the barrel and is drawn in during the dosing cycle, as shown in Figure 4. With spunbonding, the fiber webs have sufficient tear strength after calandering to be loaded using this method, however, not all non-woven materials can be, in part because of the forces needed to rotate a spool of fibers, which can be larger than 70 cm in diameter. Adding a motor to coordinate the feeding spool with the IM cycle will further improve this process. This would avoid needing to rely on the tear strength of the fabric to passively rotate the spool.

Two solid IM control samples were created; the first using the as-received pellets and the second using a spunbonded neat PP.

#### 2.5.2. Microcellular Foam Injection Molding

With the Arburg and the MuCell systems combined, high quality microcellular FIM samples can be created. A typical microcellular FIM sample comprises a uniform cellular core structure in between solid skin layers. Microcellular foams are characterized as having a minimum cell density of 10^9^ cells/cm^3^ [23]. To create IM foams, supercritical-CO_2_ (scCO_2_) is injected into the polymer melt stream as material dosing is occurring. By controlling the scCO_2_ flow rate and dosage time, the exact amount of scCO_2_ is mixed to the desired ratio. By keeping the barrel under a high backpressure, above the solubility pressure for the gas and polymer combination, a one-phase polymer and gas mixture is created. The gas is fully suffused into the polymer stream and is under a quasi-stable thermodynamic state [24]. Once the polymer and gas mixture is injected into the mold, gas nucleation will occur due to the shear and pressure drops experienced along the flow path [25]. However, by utilizing a high packing pressure after the mold filling step, prematurely formed nucleated cells can be suppressed within the mold. To initiate cell nucleation, the packing pressure is halted and the mold is retracted [26]. By retracting the mold to a fixed distance, the cavity pressure rapidly drops and the polymer undergoes a strong thermodynamic instability. As the pressure falls below the solubility pressure, cells begin to nucleate and grow [22,24,27]. The spunbond webs are fed into the Arburg injection molding machine with the same processing conditions as indicated in Table 3.

To evaluate the quality of the foam plaques with and without PET nanofibrils, the cell density and the expansion ratio were considered. To measure the cell density, a foam sample was extracted from the test plaques and cryofractured to reveal the cellular morphology. Due to the increased thickness over typical foam samples, the extracted foam sample was submerged in liquid-nitrogen for 10 min after which the samples were broken in a brittle fashion. The cryofracture samples were then loaded onto SEM stubs for viewing and sputter coating, as detailed above. SEM micrographs of the cellular morphology were viewed through the same SEM detailed above. To calculate the cell density with respect to the unfoamed volume, the following equation was used [28]:

(1) $$N_{\mathrm{unfoamed}}={\left(\frac{n_{cells}}{A}\right)}^{3/2}\times \phi$$

where $n_{cells}$ is the number of cells within a region of interest; A is the area of the region of interest; and ϕ is the expansion ratio determined by ASTM-D792. ASTM-D792 details the use of water buoyancy to evaluate the relative density of the foams. At least 100 cells were evaluated via ImageJ for each sample.

### 2.6. Mechanical Property Characterization

Testing for both the tensile and flexural properties were performed on a 3365 Universal Testing System with a 5 kN load cell (Instron, Norwood, MA, USA). For any property, five samples of each condition were tested and averaged. The mechanical test specimens can be found in Appendix A.

For both the tensile and flexural strength, the absolute strength values were calculated from the stress–strain data. However, to allow for a better comparison between the foam and solid samples, the specific strength of the material was also calculated by:

(2) $$\sigma_{specific}=\sigma_{absolute}/\rho$$

where $\sigma_{specific}$ is the specific strength; $\sigma_{absolute}$ is the absolute strength; and ρ is the density of the solid or foam sample.

#### 2.6.1. Tensile Property Characterization

After injection molding the nanofibril PET composites, the tensile samples were die cut from the test plaque. A die cutter (ODC Tooling & Molds, Waterloo, ON, Canada) was used to create the ASTM D638 Type 1 dog bone specimens [29]. To facilitate dog bone cutting, the test plaque was warmed to soften the sample and prevent cracking along the edges. The tensile tests were performed with a cross-head speed of 5 mm/min.

#### 2.6.2. Flexural Property Characterization

Unlike the tensile property testing, the flexural specimens were cut from the injection molded samples using a band saw due to their simple shape. The specimen width and support span were set relative to the sample thickness according to ASTM D790-3-point bending and are shown in Table 4 [30]. The cross-head speed was set to 5 mm/min. Testing occurred until flexural yielding, however, the data were limited to 5% strain as per the standard.

## 3. Results and Discussion

### 3.1. Nanofibril Structure of Spunbond Samples

**Figure 5:**
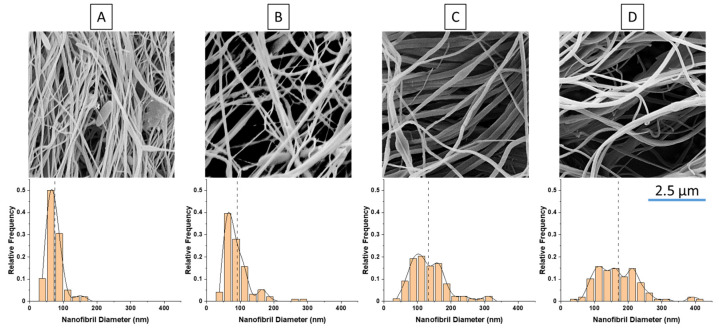
The SEM of the nanofibrils and histograms with (**A**) 1 wt% PET, (**B**) 5 wt% PET, (**C**) 10 wt% PET, and (**D**) 15 wt% PET.

Using the spunbond machine, blends of PP and PET according to Table 2 were formulated and drawn using the processing parameters in Table 1. After spunbonding, the fibers were conveyed and calandered together to create rolls of spunbond fabric. To examine how the nanofibrils behaved after spunbonding, the fibers were sectioned off and xylene was used to etch away the PP matrix. The SEM images of the nanofibril results are shown in Figure 5 [17].

**Figure 6:**
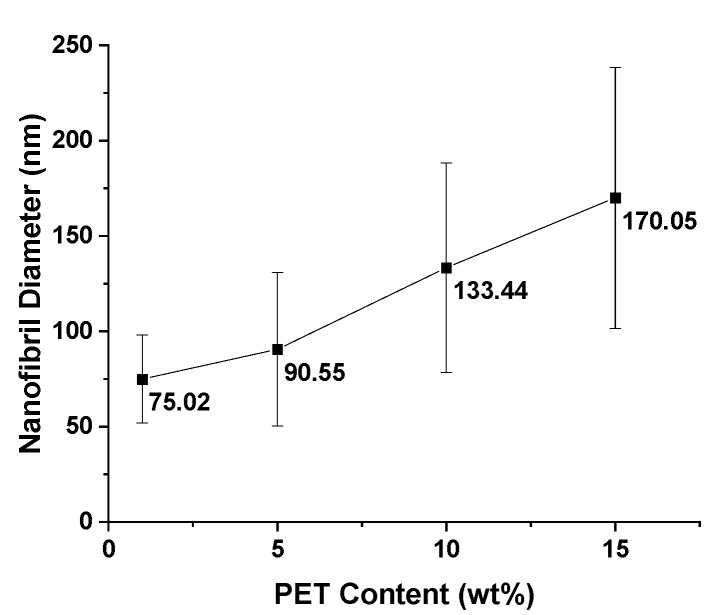
The nanofibril diameter as a function of the PET content.

The nanofibrils were measured from the SEM images to find the distribution and uniformity of the fibrils. From Figure 6, it is evident that increasing the PET content will also increase the fibril diameters, and similar ranges of fibrils have been found in other studies [2,15,18]. The increased content of PET leads to higher reinforcement, which in turns leads to increases in the viscosity and the melt strength during stretching. Having a higher resistance to stretching during spunbonding results in less deformation, which results in larger macro fibers and larger nanofibrils. Typically, it has been noted that a larger draw ratio will result in finer fibrils. The results here demonstrate that the increase in fibril diameters is due to the increase in viscosity alone, as there were no large agglomerates in the etched samples.

### 3.2. Effect of the PET Content on the Foam Structure of Foam Injection Molded Samples

**Figure 7:**
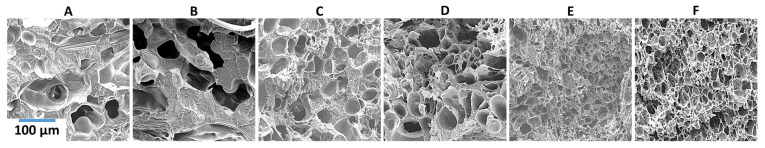
The foam SEM micrographs with (**A**) as-received PP, (**B**) 0 wt% PET, (**C**) 1 wt% PET, (**D**) 5 wt% PET, (**E**) 10 wt% PET, and (**F**) 15 wt% PET.

As shown in Figure 7, between the 0–15 wt% PET and the as-received PP, the cellular morphologies of the reinforced samples had visibly finer cells and higher uniformity. The higher melt strength and the presence of the PET nanofibrils both helped to refine the microcellular structure. As the PET nanofibrils were dispersed through the PP matrix to act as reinforcements, their presence formed a network of fibers within the PP matrix to increase the melt strength and viscosity. With a greater melt strength, the matrix is able to reduce the amount of cell coalescence after cell nucleation [27]. In addition, the PET nanofibrils will also provide increases to the cell nucleation and crystal nucleation [2,31]. The presence of the PET nanofibrils causes local pressure variations in the surrounding matrix, and this helps to lower the free energy barrier to cell nucleation. This allows for easier cell nucleation and reduces the opportunity for cells to grow instead [31]. Finally, the presence of the PET nanofibrils can positively or negatively affect the overall crystallinity and crystal sizes [32,33]. Crystals can more easily nucleate around nanofibrils [34]. Combined, this results in increased amounts of smaller crystals. The small, dispersed crystals will also apply local pressure variations and help to increase the melt strength, which increases the foam quality similarly to the nanofibrils [35].

**Figure 8:**
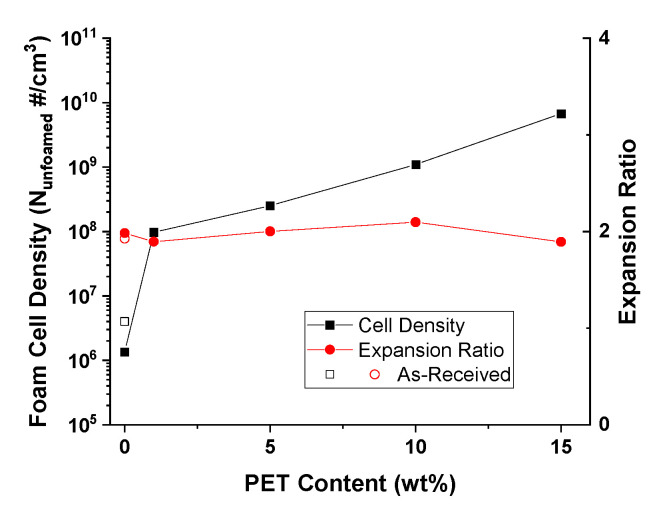
The cell density as a function of the PET content.

From Figure 8, the cell density increased with the amount of PET nanofibrils in the matrix. From the SP/99/1 sample, with a small amount of nanofibrils within the matrix, the cell density increased nearly two orders of magnitude over the SP/100/0 samples. In part, this demonstrates the effect of nanofibrils as a cell nucleating agent, as there is insufficient PET to form a fibril network. However, with an increased amount in the PET content, at 10 wt% or higher, the nanofibril reinforcement effect on the melt strength became more apparent. The cell density of the SP/85/15 sample increased by more than 3 orders of magnitude when compared to the SP/AR or SP/100/0 samples.

The expansion ratio of the foams is also presented in Figure 8. Although the FIM process was tailored to product foams at a mold opening of two times, a small variation in the expansion ratio was due to the melt strength when nucleation was induced. With a higher melt strength due to increased PET reinforcement, the SP/85/15 exhibited a small decrease in the expansion ratio compared to the other samples.

### 3.3. Effect of PET Nanofibrils on Crystallization Behavior

**Figure 9:**
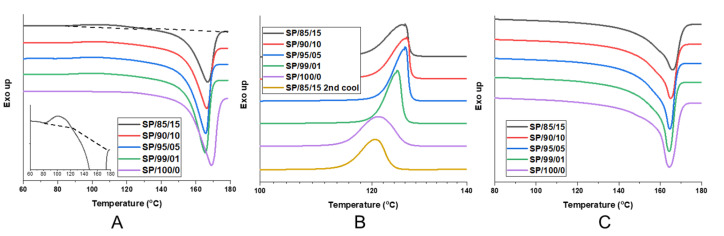
The DSC thermograms of the PP and PET composites after spunbonding: (**A**) first heating (inset shows SP/85/15 with PET cold crystallization peak; the dashed line indicates the baseline), (**B**) first cooling plus SP/85/15 s cooling, and (**C**) second heating.

**Figure 10:**
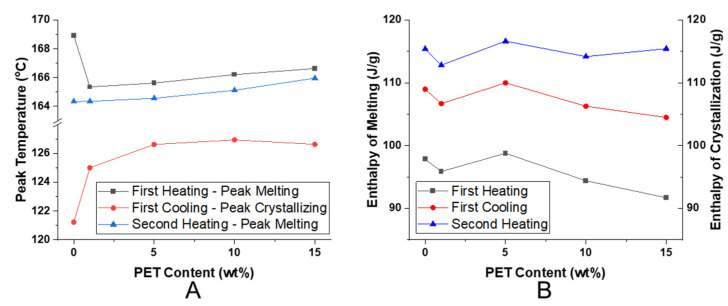
The analysis of the DSC thermograms: (**A**) peak melting and crystallizing temperatures, (**B**) enthalpy of melting and crystallization.

The DSC characterization results of the spunbonded PP with nanofibrillated PET are shown in Figure 9 and are summarized in Figure 10. The PP melting and crystallizing enthalpies were normalized by the weight fraction of PP within the composite.

From the first heating cycle in Figure 9A, the neat SP/100/0 had the highest peak melting temperature. This was followed by a sharp decrease of 3.5 °C with SP/99/01, after which there was a small increasing trend with the PET content. The decrease seen in SP/99/01 may be due to a decreased orientation leading to decreased crystallization during spunbonding with the inclusion of the PET nanofibrils [36]. With an increase in the amount of PET nanofibrils between SP/99/01 and SP/85/15, it appears that the peak melting temperature increased slightly, however, the melting enthalpy decreased. Between the first and second heating, there was a clear difference in the melting behavior. The elevated peak melting temperature for SP/100/0 was no longer evident in the second heating. The orientation from spunbonding was no longer present during the second heating, and as a result, a lower peak melting temperature was seen for all samples. 

A small exothermic shoulder was evident for all of the PET composites immediately before the melting peak. The size of the exothermic peak grew with the PET loading, and was most visible in the SP/85/15 sample, as seen in the inset of Figure 9A. The SP/85/15 heating curve demonstrates the shoulder between 80 and 120 °C. It is possible that this phenomenon is in part related to the cold crystallization of PET [37,38]. As PET is a slower crystallizing polymer relative to PP, it may be possible that the PET nanofibrils are oriented and frozen during the spunbond process, as such, they are not fully crystallized. Another possibility is that the already nucleated PP crystals are changing or growing at this temperature. This phenomenon was not seen during the second heating where the samples experienced a slower cooling rate, allowing more time for crystallization to occur (Figure 9C) during the spunbonding process where the extrudate rapidly cools from 260 to 30 °C (>100 °C/s); however, during the first cooling, there was sufficient time for crystallization to occur (~0.17 °C/s).

From Figure 9B, the presence of the PET nanofibrils made a significant impact on the crystallization kinetics during the first cooling. For samples SP/95/5 through SP/85/15, the crystallization peak occurred earlier at higher temperatures and was faster. This was evidenced by the sharp increase in the heat flow on the right hand of the peak. The initial slope of the crystallization peak has been linked to the crystal nucleation rate [39,40]. Accordingly, the peak crystallization temperature increased with the PET nanofibril loading, as seen in Figure 10A. With the presence of the PET nanofibrils, the PP chains were less mobile and formed into crystals at a temperature up to 5.5 °C earlier compared to SP/100/0. The SP/100/0 sample demonstrated a uniform crystallization peak with a gentle rise and fall. To see the effect of the PET nanofibrils, a second cooling was performed on SP/85/15 after it was heated to 280 °C. At this temperature, the PET nanofibril structures will be erased and return to spherical domains. As a result, during the second cooling, the PP crystallization peak was similar to the neat SP/100/0 sample. Therefore, the shape of the PET nanofibrils has a strong effect upon the crystallization kinetics and behavior.

When the enthalpy of melting for the first and second heating cycles were compared in Figure 10B, the first heating enthalpy values were lower and trended downward. The first heating occurred after spunbonding, where the PP and PET nanofibrils experienced high orientation and fast cooling, which limited the amount of time for PP crystal growth. Similarly, the addition of increased PET content appears to be a negative influence. However, for the second heating, the slower cooling rate allowed for more crystal structures to develop, resulting in a flat trend for the melting enthalpy. From Figure 10B, the PET content did not appear to affect the overall amount of the crystals developed. However, it is likely that the earlier crystallization kinetics actually change the PP crystal structures. 

The faster crystallization kinetics seen during the first cooling will be similar to what is occurring within the injection molding process. Within the core of the injection mold, the polymer and gas mixture will cool at a slower rate (~3.33 °C/s) to the mold temperature within the dwelling time [41]. The addition of CO_2_, which acts as a plasticizer, will increase the crystallization kinetics. The faster kinetics help to improve the foam cellular morphology [42,43].

### 3.4. Mechanical Properties

#### 3.4.1. Tensile Properties of Injection Molded PP/PET-Nanofibril Composites and Foams

##### Tensile Properties of Solid Injection Molded PP/PET-Nanofibril Composites

**Figure 11:**
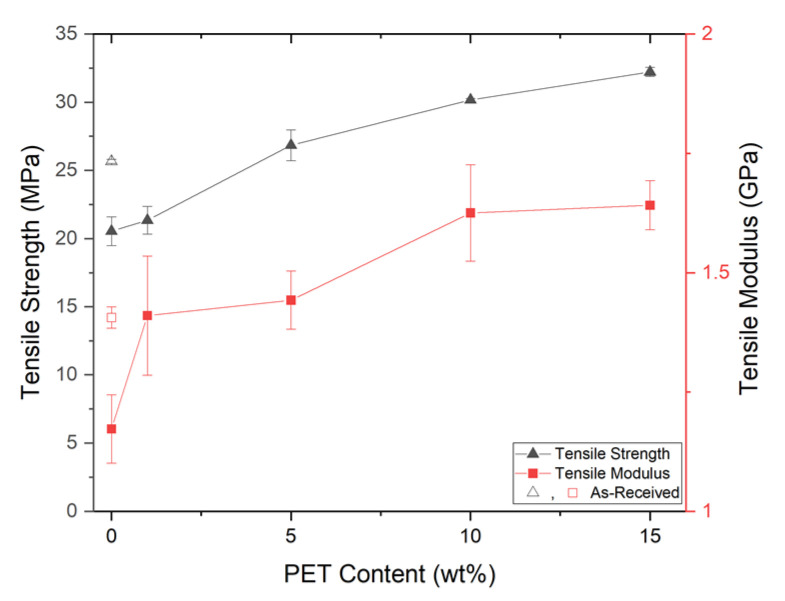
The solid tensile properties.

After injection molding the solid and foam PET nanofibrils composites, tensile dog bone specimens were cut from the test plaques using a die (see Section 2.5.1). From Figure 11, increasing the amount of PET reinforcement also improved the overall tensile strength. When comparing the SP/100/0 and SP/99/1 samples, there were only very slight gains in the tensile strength but more significant gains in the tensile modulus. This is likely to be because a network of PET fibrils has not been established [2]. However, when a nanofibril network is established within the matrix, the tensile strength of the composites begins increasing with the PET loading level. With SP/85/15, SP/90/10, and SP/95/5, the tensile strength increased by 57, 47, and 31%, respectively, as seen in Table 5. When compared to SP/100/0, there was a notable jump in the tensile modulus with SP/99/1, however, a jump in tensile strength was not seen until SP/95/5. These results demonstrate improved tensile properties over the SP/100/0 control sample; however, in comparison to the SP/AR control, the improvements were modest. When considering the SP/AR control, more than 5 wt% PET was required to see positive effects (i.e., SP/90/10) for either the strength or modulus.

At 15 wt% PET, relative increases in the tensile strength results appeared to align with the highest drawing ratio samples of [44] and outperformed the nanofibrils found in [15]. The results presented here also demonstrated higher increases in the tensile strength when compared to spherical blends of PP/PET at higher loadings [45]. This indicates that the PET fibrillar morphology is vital to achieving higher tensile strength.

#### Tensile Properties of Foam Injection Molded PP/PET-Nanofibril Composites

**Figure 12:**
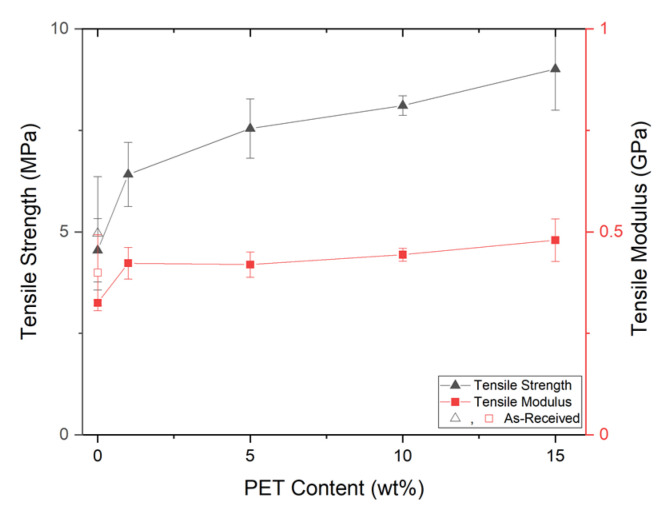
The foam tensile properties.

With FIM, the foam structures seen in Figure 7 were created and dog bone specimens were die cut and tested. Similar to the solid samples, the foam tensile strength increased regardless of the amount of PET nanofibrils included with respect to both SP/100/0 and SP/AR. The improved foam tensile properties in Figure 12 can be attributed to two factors: the PET reinforcement and the microcellular morphology. The increase in the SP/99/1 sample was primarily attributed to the improved cellular morphology as there was not a significant amount of reinforcement with only 1 wt% PET. For the SP/95/5 and above samples, the increased matrix reinforcement and smaller cell sizes both helped to increase the foam tensile strength. Similar results were seen in a system using PLA/PET [22]. As seen in Table 6, the PET loading had a stronger effect upon the foam tensile strength than the foam tensile modulus.

**Figure 13:**
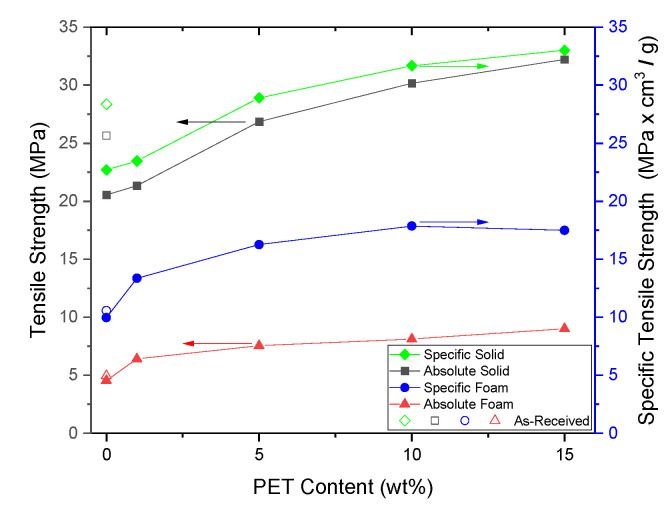
The specific tensile strength of the solids and foams.

When the specific strength of the foam and solid materials were taken into account, the foamed samples did not demonstrate the same level of strength as their solid counterparts, as seen in Figure 13. However, the specific strength of the foamed SP/90/10 and SP/85/15 samples were approximately 78% of the solid specific strength. This was a positive result as there was a 50% reduction in density.

### 3.4.2. Flexural Properties of Injection Molded PP/PET-Nanofibril Composites and Foams

#### Flexural Properties of Solid Injection Molded PP/PET-Nanofibril Composites

**Figure 14:**
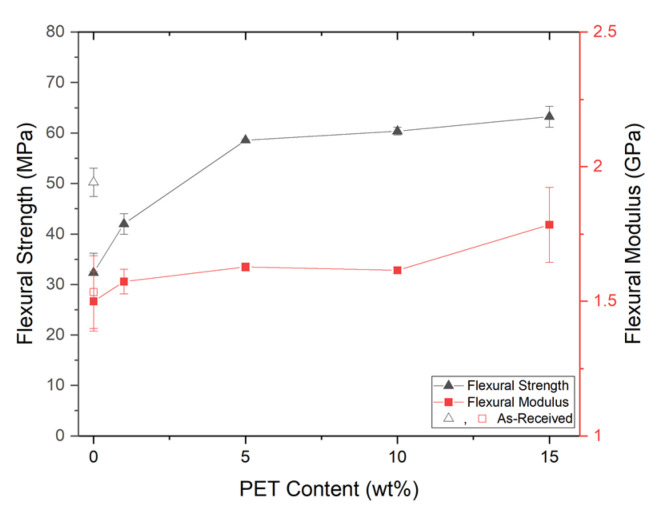
The solid flexural properties.

A similar trend to the solid tensile properties was seen with the solid flexural properties. From Figure 14, at a low loading of PET nanofibrils (SP/99/01), there was a modest increase in the flexural strength. However, when the PET loading was increased to 5 wt% (SP/95/05), the flexural strength exhibited a greater increase of up to 80%. Beyond 5 wt%, the flexural properties appeared to exhibit a plateau as the flexural properties did not significantly increase. When comparing the behavior of SP/95/05 to SP/85/15, the improvement in flexural strength was lower than between SP/99/01 and SP/95/05.

When performing the flexural testing, samples SP/85/15 through SP/95/05 did not break/fracture at the 5% strain limit, as indicated in Table 7. Conversely, the unreinforced SP/AR and SP/100 both broke at the 5% strain limit, as did SP/99/01. This indicates that with sufficient loading, the PET nanofibrils are effective at reinforcing the PP matrix. The PET nanofibrils increased the flexural strength and, subsequently, the fracture behavior. A similar behavior was also seen in [10]. However, other research has identified lower improvements at higher PET loadings, which may indicate excessive PET loading to be detrimental [46]. From another study, the addition of a compatibilizer can lead to results better than the rule-of-mixtures would predict [45].

#### Flexural Properties of Foam Injection Molded PP/PET-Nanofibril Composites

**Figure 15:**
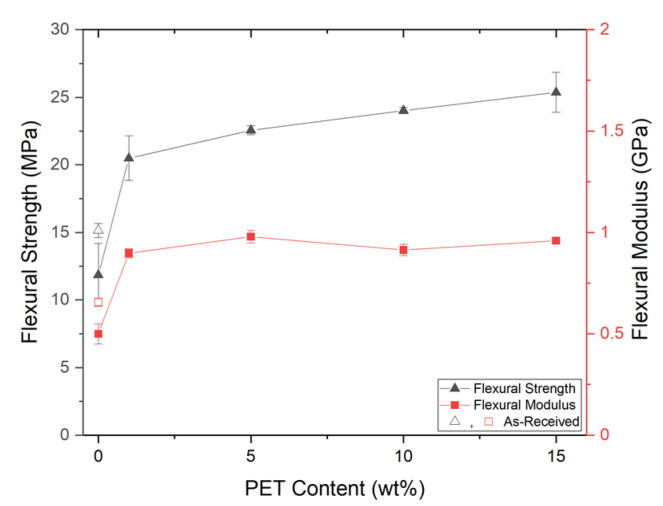
The foam flexural properties.

With foaming and nanofibril reinforcement, the samples demonstrated improved flexural properties. From Figure 15, with SP/90/10 and SP/85/15, there were two-fold improvements in both the flexural strength and modulus. The SP/95/05 sample saw improvements in the flexural strength and similar modulus. As with the solid properties, the foam reinforcement appeared to reach a plateau as the PET content is increased from 1 to 15 wt%. A direct effect from the foam structure can be seen between the SP/99/01 and SP/100/0 samples.

Using the 1 wt% PET as a cell nucleating agent, the cell density was increased and the foam structure became more uniform, as seen in Figure 7 and Figure 8. The foam flexural properties can be directly related to these improvements in the foam structure. At the 5% strain limit, none of the foam samples broke except for the SP/95/05 sample, indicating differing failure mechanisms, as shown in Table 8. It is possible that with a lower amount of PET reinforcement (<5 wt%), the sample will remain ductile and flexible due to the cellular morphology of the foams of SP/99/01 and below. However, when the amount of PET reinforcement increases, the stiffness of the samples also increases. With SP/90/10 and above, the high degree of reinforcement increased the flexural strength of the samples, preventing them from failing. However, in-between these two mechanisms (SP/95/05), the increased strength reduced the foam’s flexibility, but did not impart enough flexural strength to prevent failure.

**Figure 16:**
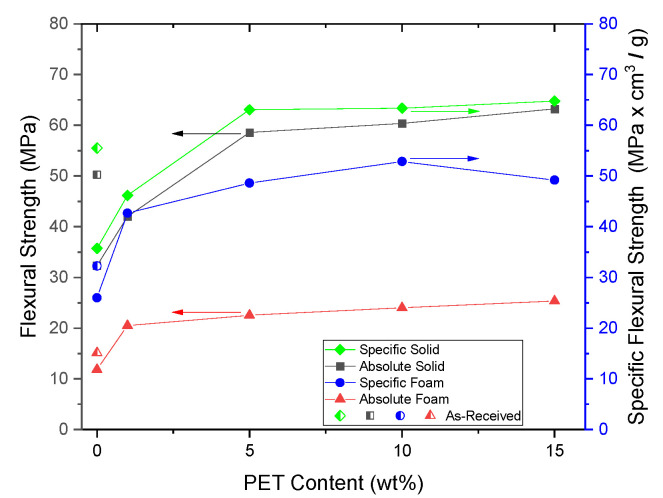
The specific flexural strength of the solids and foams.

The specific flexural strength and modulus of the solid and foam samples are shown in Figure 16. For the SP/90/10 specimens, due to the improved foam quality and the PET nanofibril reinforcement, the specific strength of the foam was nearly equal to the as-received solid samples. This indicates that even with a 50% reduction in weight, similar properties can be achieved by the addition of 10 wt% PET nanofibrils. The specific strength of the SP/85/15 samples decreased slightly due to the decreased expansion ratio.

## 4. Conclusions

Nanofibril PET and PP composites were successfully produced and tested using continuous processing equipment. The PET nanofibrils were created by applying a high extensional stress on a blend of PP and PET using a spunbond machine. Depending on the loading of PET, nanofibrils between 75 and 170 nm were formed. As the PET loading increased, so did the nanofibril diameter. After calandering the spunbond webs, the spools of composite materials were loaded directly into an injection molding machine. From the injection molding machine, solid and foam specimens were created. With nanofibril PET reinforcement, the cell density was increased by more than three orders of magnitude. Using both the solid and foam samples, the tensile and flexural mechanical properties were tested. With increasing PET reinforcement, both the solid tensile and flexural properties were noticeably improved. The solid tensile properties were improved by 50%, however, the foam strength increased by 100%. For the flexural strength in both the solid and foam samples, there was a 100% increase. When the specific flexural strengths were taken into account, the foam samples with a 10 wt% PET loading were similar to the solid as-received PP samples.

## Figures and Tables

**Table 1:** The spunbond processing parameters.

Process Parameter	Process Value
Extruder temperature	260 °C
Die temperature	260 °C
Spinneret die diameter	300 μm
Spinneret die land	5 mm
Spinneret die count	90 holes
Drafting pressure	0–15 psi

**Table 2:** The spunbond samples and identification.

Material Code	PP Content (wt%)	PET Content (wt%)
SP/85/15	85	15
SP/90/10	90	10
SP/95/05	95	5
SP/99/01	99	1
SP/100/0	100	0
SP/AR	As-Received	---

**Table 3:** The injection molding processing parameters.

Process Parameter	Process Value
Injection speed	100 cc/s
Extruder temperature	180 °C
Mold temperature	80 °C
Screw speed	50 rpm
CO_2_ content	10 wt%
Mold opening distance	2×
Mold opening time	20–30 s

**Table 4:** The flexural specimen dimensions.

Sample	Thickness (mm)	Width (mm)	Length (mm)	Span (mm)
Solid	3	12	65	48
Foam	6	24	130	96

**Table 5:** The solid tensile improvement relative to SP/100/0 and SP/AR.

	Material Code	Strength	Modulus
**To SP/100/0**	SP/85/15	56.8%	40.0%
	SP/90/10	46.9%	38.6%
	SP/95/05	30.7%	23.0%
	SP/99/01	3.9%	20.3%
**To SP/AR**	SP/85/15	25.5%	16.7%
	SP/90/10	17.6%	15.6%
	SP/95/05	4.6%	2.6%
	SP/99/01	−16.8%	0.3%

**Table 6:** The foam tensile improvement relative to SP/100/0 and SP/AR.

	Material Code	Strength	Modulus
**To SP/100/0**	SP/85/15	98.4%	47.5%
	SP/90/10	78.5%	36.5%
	SP/95/05	66.0%	29.0%
	SP/99/01	41.2%	30.0%
**To SP/AR**	SP/85/15	81.6%	20.1%
	SP/90/10	63.5%	11.2%
	SP/95/05	52.0%	5.1%
	SP/99/01	29.3%	5.9%

**Table 7:** The solid flexural improvement relative to SP/100/0 and SP/AR.

	Material Code	Strength	Modulus	Fracture?
**To SP/100/0**	SP/85/15	95.56%	18.96%	No
	SP/90/10	86.70%	7.71%	No
	SP/95/05	81.18%	8.52%	No
	SP/99/01	29.88%	4.89%	Yes
**To SP/AR**	SP/85/15	25.89%	16.31%	No
	SP/90/10	20.18%	5.30%	No
	SP/95/05	16.62%	6.10%	No
	SP/99/01	−16.39%	2.55%	Yes

**Table 8:** The foam flexural improvement relative to SP/100/0 and SP/AR.

	Material Code	Strength	Modulus	Fracture?
**To SP/100/0**	SP/85/15	113.99%	91.93%	No
	SP/90/10	102.62%	82.80%	No
	SP/95/05	90.32%	95.88%	Yes
	SP/99/01	72.84%	79.54%	No
**To SP/AR**	SP/85/15	67.51%	46.21%	No
	SP/90/10	58.61%	39.25%	No
	SP/95/05	48.98%	49.22%	Yes
	SP/99/01	35.30%	36.77%	No

**Figure A1:**
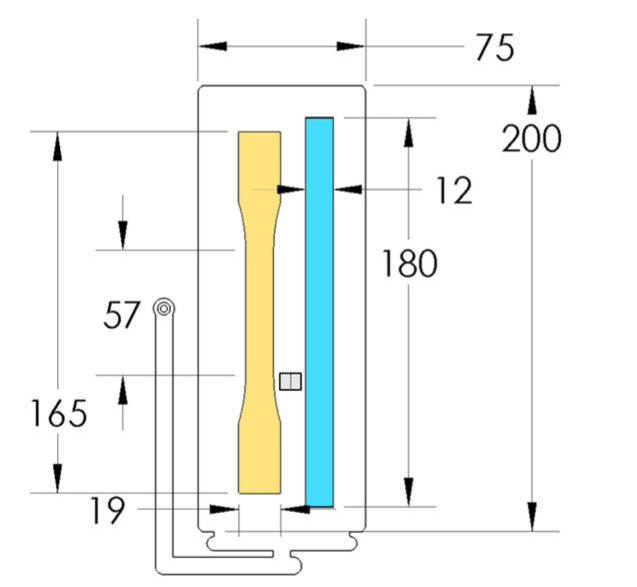
The dimensions of the injection molded plaque and mechanical testing specimen in mm.

## Data Availability

Not applicable.

## Appendix A

Figure A1 indicates the nominal dimensions of the plaque for solid and foam injection molding. Figure A1 also shows the size of the tensile and flexural specimens. Figure A1 also shows the SEM sample location, with the SEM fracture surface parallel to the injection flow direction.
